# Inhalable Nano Formulation of Cabazitaxel: A Comparative Study with Intravenous Route

**DOI:** 10.1002/mabi.202400567

**Published:** 2025-01-30

**Authors:** Elif Kaga, Sadik Kaga, Korhan Altunbas, Nurullah Okumus

**Affiliations:** ^1^ Department of Medical Services and Techniques Afyonkarahisar Health Sciences University Afyonkarahisar 03030 Türkiye; ^2^ Department of Biomedical Engineering Afyon Kocatepe University Afyonkarahisar 03200 Türkiye; ^3^ Department of Histology and Embryology Afyon Kocatepe University Afyonkarahisar 03200 Türkiye; ^4^ Department of Pediatrics Afyonkarahisar Health Sciences University Afyonkarahisar 03030 Türkiye

**Keywords:** cabazitaxel, drug delivery, inhalation, polymeric nanoparticle, toxicity

## Abstract

Chemotherapy is generally given by intravenous (IV) administration which provides higher bioavailability than other systemic routes. However, in the case of lung cancer, the pulmonary (INH) route is the other choice for inhalable formulations. In the study, biochemical and histological parameters of Cabazitaxel (CBZ) free (2 mg kg^−1^) and nanoparticle (NP) (2 mg kg^−1^ CBZ equivalent) formulations are investigated after IV and INH administration in rats. The nanoformulation of CBZ is obtained using PEGylated polystyrene (PEG‐PST) nanoparticles obtained by PISA. While a nose and head‐only device is used for INH administration, a jugular vein is used as the IV route. Blood samples (blank, 24 h, and 48 h) are collected via carotid artery cannulas without handling in metabolism cages. According to biochemical parameters, free CBZ formulation applied via IV or INH route shows higher systemic toxicity. On the other hand, the nanoformulation of CBZ showed no signs of toxicity in both IV or INH routes. Higher and longer retention is observed in the case of inhaled nanoformulation. Histological analysis showed higher alveolar macrophage migration for inhaled nanoformulation due to enhanced retention. Results showed that nanotechnology and the lung defense system gave the advantage to end up with an inhalable nanomedicine formulation for lung cancer.

## Graphical abstract

Nano drug formulation shows no signs of toxicity for IV and INH routes, whereas higher systemic toxicity occurs in free drug formulation in both. Inhalable nano formulation provides higher and longer retention in deep lung regions due to its size and defies rapid systemic absorption in the lungs. Inhalable nanodrug formulations have much promise for lung‐targeted chemotherapy.

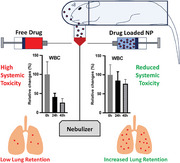

## 1 Introduction

Cancer cells are physiologically different than healthy cells due to abnormal gene mutations. These genetic anomalies cause various physiological changes such as cell heterogeneity in solid tumors. Cell heterogeneity in the tumor tissue causes the molecular mechanism to change against chemotherapy drugs in conventional oncotherapy. For example, suboptimal drug concentrations in tumor tissue may exhibit poor therapeutic efficacy causing drug resistance.^[^ ^1^ ^]^ In addition to multidrug resistance, obstacles such as low cure rates, high drug toxicity, and long‐term treatment regimens are the main factors in cases where chemotherapy fails.

Chemotherapy drugs must reach cancer tissues in the required concentrations for an effective antitumoral treatment. Intravenous (IV) administration is the most common route for chemotherapy. However, it inevitably causes a large proportion of chemotherapeutics to be widely distributed in various organs and leads to accumulation in tumor regions at significantly low drug concentrations. That generally occurs due to several factors such as presystemic metabolism, large body surface area (BSA), and nonspecific distribution.^[^ ^2^ ^]^

Localized chemotherapy aims to deliver anticancer drugs directly to cancerous tissues. This allows higher drug concentrations in the tumor tissue compared to nontargeted tissues.^[^ ^3^ ^]^ Although localized chemotherapy is not possible for all cancer types at desired levels via common administration routes, inhalable drug formulations have a great potential to cargo the desired agents into the lungs via inhalation. Inhalation is a noninvasive drug administration route for both systemic and local drug delivery that targets the respiratory system thanks to the lung's distinct properties such as large surface area and high permeability as well as being a major port of entry.^[^ ^4^ ^]^ Studies have shown that inhalation treatments are more effective than systemic treatments in certain diseases. These treatment strategies are usually for respiratory diseases such as asthma, chronic obstructive pulmonary disease (COPD), and respiratory tract infections.^[^ ^5^ ^]^ Inhalation is a promising method in local drug treatment of lung cancer apart from these respiratory system diseases.^[^ ^6^ ^]^

As well as being a noninvasive route of administration INH possesses crucial advantages, such as avoiding first‐pass effect and low metabolic activity compared to other elimination organs. Despite these advantages, most inhalable drug formulations don't have an ideal half‐life and bioavailability at the lung. There are important challenges to achieving effective local pulmonary therapy mainly, rapid systemic absorption, alveolar macrophage clearance, mucociliary clearance, and enzymatic degradation.^[^ ^7^ ^]^

Nano and micro drug delivery formulations can overcome these challenges thanks to their macromolecular size, controllable drug release profiles, and ability to escape from pulmonary defense mechanisms via surface modification.^[^ ^7^, ^8^ ^]^ Small active agents suffer from rapid systemic absorption due to their size, alveolar macrophage clearance due to their foreign structure, and enzymatic degradation due to the lack of a firewall. Therefore, size and structure properties are the basic parameters that must be considered for pulmonary‐targeted nanoformulations to demonstrate their efficiency. The nanoparticles must be big enough to penetrate lung tissues and prevent rapid exhalation.^[^ ^9^ ^]^ In addition to escaping from the pulmonary defense mechanism, another advantage of drug loading into a nanoparticle is reducing the lung tissue surface area exposed to the drug per unit time. This allows the targeted tissue to be exposed to the active agents for a long time while reducing the amount of drug entering the systemic circulation over time. Thus, in addition to providing more effective treatment, it also reduces the side effects in nontargeted tissue or organs.^[^ ^10^ ^]^

There are four main types of inhalation devices; dry powder inhalers (DPIs), pressurized metered‐dose inhalers (pMDIs), soft mist inhalers (SMIs), and nebulizers. The chemical structure and solubility of the active agent and vehicle are the major parameters for determining the preparation of the inhalable formulation and device to use.^[^ ^11^ ^]^

Inhalable nanoformulations of chemotherapy drugs such as Cisplatin, 5‐FU, Doxorubicin, Gemcitabine, Carboplatin, Docetaxel, Paclitaxel, and many others have been widely investigated for lung cancer. Liposomes, micelles, dendrimers, polymeric nanoparticles, lipid nanoparticles, and polymer‐drug conjugates have been widely used as nano vehicles in this area.^[^ ^7^, ^11^, ^12^ ^]^ Thanks to the efforts of nano‐formulated inhalers, there have been new inhalable nano‐drug candidates for lung cancer in clinical trials.^[^ ^13^, ^14^, ^15^ ^]^

The main reason for the side effects resulting from traditional systemic chemotherapy is the toxicity in nontarget tissues as a result of nonspecific biodistribution. However, many chemotherapy drugs can still be licensed due to the risk‐benefit assessment. Although inhalable nanoformulations are suitable for lung targeting, a certain amount of retention may occur in the throat/larynx in the respiratory tract due to the lung defense mechanism. However, the potential toxicity that may occur is quite low potential compared to the direct exposure to systemic circulation caused by IV chemotherapy administration. Because mucociliary clearance^[^ ^7^ ^]^ is the first step of the lung defense mechanism to eliminate the structures captured here. Thereby, since the chemotherapy agent is inactive in the nanoparticle and will pass into the stomach through mucosal fluids and be metabolized, the toxicity created in the throat/larynx region will be limited.

In this study, polymeric PEG‐PST nanoparticles were fabricated using the polymerization‐induced self‐assembly (PISA) technique.^[^ ^16^, ^17^ ^]^ The shell layer of this two‐layered nanostructure consists of Poly(ethylene glycol) methyl ether methacrylate (PEGMEMA) and the core consists of polystyrene (PST). PEGMEMA^[^ ^18^, ^19^, ^20^ ^]^ and PST^[^ ^21^, ^22^, ^23^ ^]^ are widely used polymers in nanodrug formulations.

Core/shell‐structured this nanoparticle bears several advantages. The hydrophilic PEGMEMA shell with its branched polyethylene glycol (PEG) structure provides the water stability of the PST nanoparticle core by disabling aggregate formation. The hydrophobic PST core segment allows the loading of the hydrophobic drugs or tag molecules (CBZ and NR for this study). Moreover, as shown in the previous study^[^ ^25^ ^]^ the PISA approach to obtain PEGMEMA‐PST‐based core/shell‐structured nanoparticles enables to get nanoparticle solutions with high concentration. Also, since the PST core was self‐covered with a branched PEG shell no further surface modification or any stabilizer usage is required.

Within the scope of the study, besides the nano CBZ formulation, a free CBZ formulation was also prepared. After drug release and cytotoxicity studies on the A549 cell line (human lung carcinoma), these formulations were applied to catheterized rats through IV and INH routes. For the INH route, nebulized free and nano CBZ formulations were applied to rats using nose and head‐only device apparatus. The toxicological and histological evaluation of the INH application of the developed nano CBZ formulation compared to both free CBZ and IV application was made with tissue and blood samples taken from rats in metabolism cages.

## 2 Results and Discussion

### 2.1 Synthesis of Nanoparticles via PISA

**Scheme 1:**
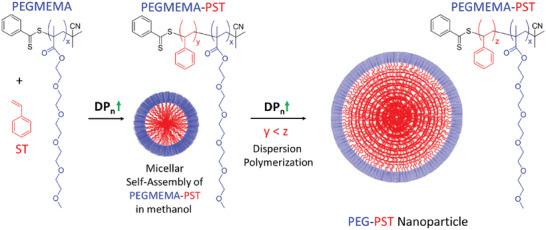
A schematic illustration of the nanoparticle formation stages.

PEGMEMA polymer as macro‐CTA was synthesized by RAFT polymerization according to previously reported literature procedure^[^ ^25^ ^]^ as detailed in the method section. PEGMEMA homopolymer with a molecular weight (Mn) of 7.400 g mol^−1^ and a PDI value of 1.08 (yield: 85%) after 16 h of polymerization. PEGMEMA polymer with such molecular weight (7.400 g mol^−1^) has ≈25 repeating units since a monomer (OEGMEMA) with a molecular weight of 300 g mol^−1^ was used. The molecular weight (7.400 g mol^−1^) of PEGMEMA macro‐CTA was suitable for PISA nanoparticle synthesis to get stable nanoparticles with a wide hydrophilic shell when compared with familiar PEGylated PST nanoparticles.^[^ ^24^, ^34^, ^35^ ^]^ To obtain spherical PEG‐PST nanoparticles, the PISA dispersion polymerization was performed using ST monomer and PEGMEMA macro‐CTA in methanol as detailed in the method section. In the PISA approach, insolubility of the PST segment in methanol induces self‐assembly of newly formed PEGMEMA‐PST block copolymers, and the degree of polymerization (DP_n_) increases in dispersion conditions.^[^ ^34^, ^35^ ^]^ **Scheme** **1** shows a schematic illustration of the PEG‐PST nanoparticle formation stages.

**Figure 1:**
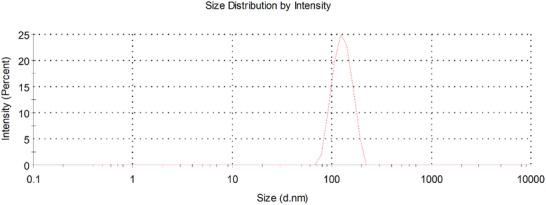
Hydrodynamic size distribution of PEG‐PST nanoparticles. Size distribution was calculated as intensity‐based using DLS.

The milky white and turbid reaction solution was observed after 12 h of polymerization as evidence of nanoparticle formation. The concentration of purified nanoparticle solution was calculated as 26 mg mL^−1^. The hydrodynamic diameter of prepared nanoparticles was measured using DLS. The intensity‐based size distribution of nanoparticles is shown in **Figure** **1**. The hydrodynamic diameter of nanoparticles was measured as 121.7 nm (STD: 27.1 nm) with an average PDI value of 0.033.

**Figure 2:**
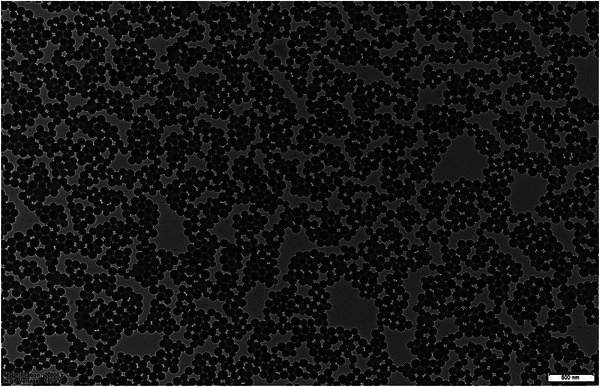
TEM image of spherical PEG‐PST nanoparticles (acceleration voltage of 100 kV, at magnifications of 20 000, scale bar: 500 nm).

The TEM image of synthesized nanoparticles is shown in **Figure** **2**. According to TEM analysis, it is clear that uniform and spherical PEG‐PST nanoparticles with dimensions of ≈100 nm were synthesized.

### 2.2 Nile Red (NR) and CBZ Loading

To carry out cell internalization and lung penetration studies nanoparticles were loaded with NR, whereas they were loaded with CBZ for cytotoxicity studies and biochemical evaluation. A gradient dialysis medium was used to slowly increase the hydrophilicity of the medium while preventing the precipitation of hydrophobic NR and CBZ during loading. The nanoparticle samples in methanol solution were dialyzed against methanol/water mixtures with increasing water ratios at each medium change stage. Since prepared nanoparticles are stable in both water and methanol^[^ ^34^, ^35^ ^]^ and NR and CBZ are insoluble in water, a methanol‐to‐water gradient was used for the post‐synthesis drug loading approach. Thanks to this gradient‐based strategy, hydrophobic molecules (CBZ or NR) were accumulated in the hydrophobic PST core of PEG‐PST nanoparticles without precipitation due to the decreasing hydrophobic character of the dialysis medium over time. Loading efficiency for CBZ and NR was calculated as 95% and 99%, respectively. As a result, 9.9% CBZ‐loaded and 1.9% NR‐loaded PEG‐PST nanoparticle solutions were obtained. Loading parameters for CBZ‐loaded and NR‐loaded PEG‐PST NPs are summarized in **Table** **1**.

**Table 1:** Loading parameters for CBZ‐loaded PEG‐PST (CBZ‐PEG‐PST) and NR‐loaded PEG‐PST (NR‐ PEG‐PST) nanoparticles.

	The initial mass of loaded material	NP mass	Loading efficiency [LE]	Loading content [LC]
CBZ‐PEG‐PST	18 mg	156 mg	95%	9.9%
NR‐PEG‐PST	3 mg	156 mg	99%	1.9%

Although postsynthesis drug loading approaches seem unfavorable compared to one‐pot drug loading and synthesis strategies for loading efficiency, CBZ‐PEG‐PST and NR‐PEG‐PST nanoparticles were obtained with high loading efficiency values due to the gradient dialysis‐based postsynthesis approach. The intended level of NR and CBZ loading capacities has been achieved. Since 1.9% NR loading capacity was sufficient as a tag molecule considering cell internalization and lung penetration studies and 9.9% CBZ loading capacity was optimal for cytotoxicity studies and biochemical evaluation, no other dose formulations were prepared.

### 2.3 Release Profiles of NPs

**Scheme 2:**
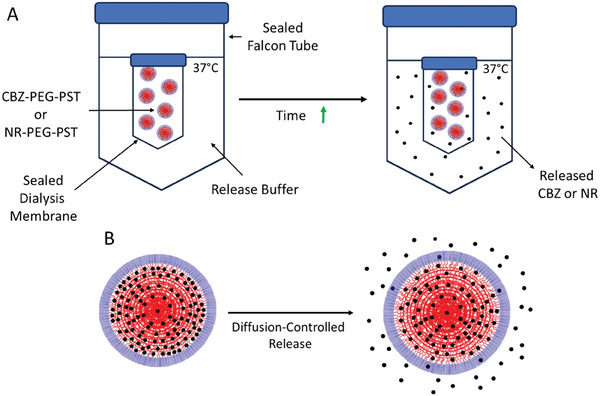
The schematic illustration of the release study A) and the release mechanism of CBZ or NR‐loaded PEG‐PST nanoparticles B).

The release study was performed to understand the ability of the nanoparticles to carry hydrophobic load in physiological conditions. Actual CBZ and NR release profiles of nanoparticles were determined using calibration curves of each at 277 and 532 nm, respectively. As a common procedure ethanol and Tween 80 were added to the release buffer to eliminate precipitation of released CBZ or NR in PBS buffer (pH: 7.4).^[^ ^36^ ^]^ A schematic illustration of the release study and the release mechanism of CBZ or NR‐loaded PEG‐PST nanoparticles is shown in **Scheme** **2**.

**Figure 3:**
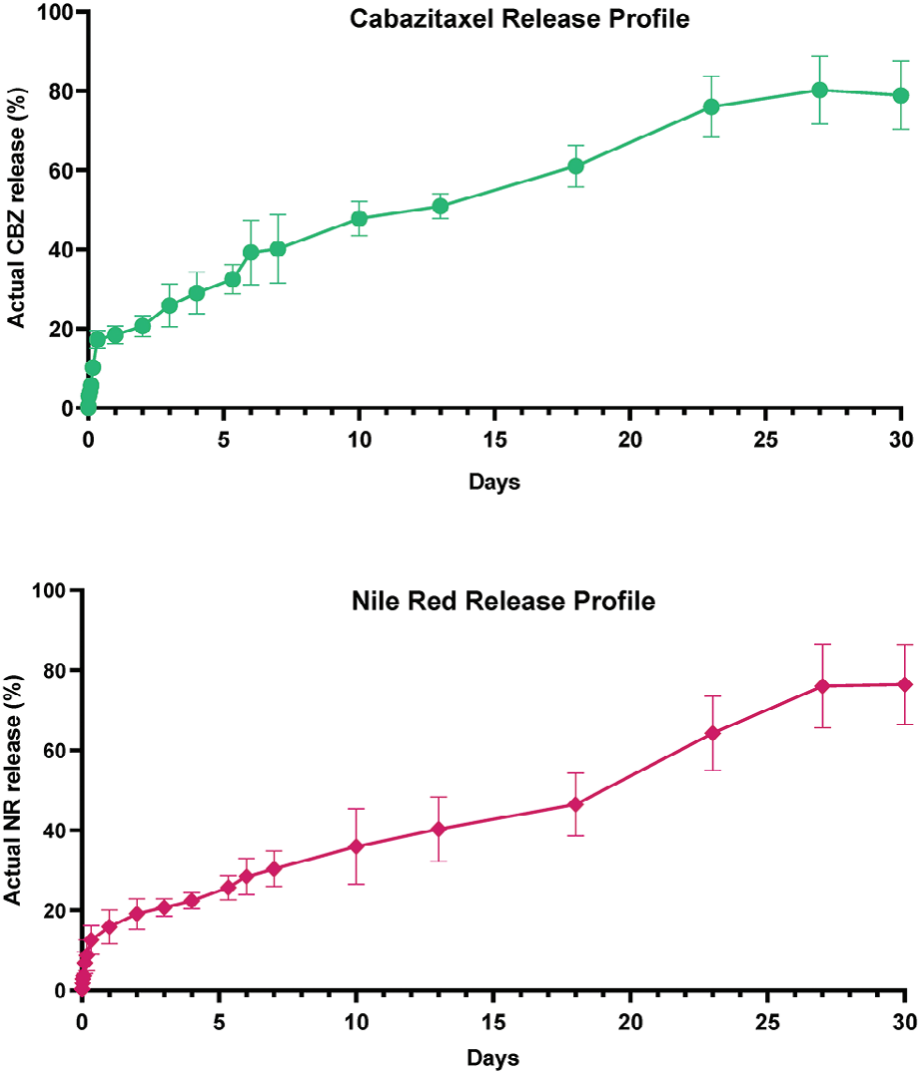
The release profiles of the CBZ‐PEG‐PST and NR‐PEG‐PST. The dialysis unit was used to perform release studies in phosphate buffer saline (PBS) (0,1% tween 80, 10% ethanol, pH: 7.4), and the data is presented as a percentage of actual CBZ and Nile Red release. Each point was represented in mean ± SD (*n* = 3).

Polymeric nanoparticles have various drug release mechanisms such as erosion‐controlled, osmotic pumping‐controlled, diffusion through water‐filled pores, and diffusion through the polymer matrix.^[^ ^37^ ^]^ Release studies revealed that PEG‐PST nanoparticles didn't show burst release. They showed prolonged release profiles in aqueous media due to the diffusion‐controlled release through the polymer matrix. The results were similar in both cases where CBZ and NR release were analyzed as shown in **Figure** **3**.

CBZ‐PEG‐PST released 50% of loaded CBZ in ≈5 days and a total of 75% CBZ release was achieved on the day of 15. Similarly, NR‐PEG‐PST released 50% of loaded NR in ≈5 days and a total of 75% CBZ release was achieved on the day of 15. The nanocarriers with stable architecture and sustained release profiles are preferred when both physiological monitoring of the loaded molecule and physiological stability in circulation are aimed.^[^ ^38^ ^]^ The fact that many CBZ and NR loaded micellar type nanocarriers in the literature^[^ ^39^, ^40^, ^41^ ^]^ reach these values in hours under similar conditions reveals the suitability of the PEG‐PST structure in demonstrating the proof‐of‐concept in this study. It is probably due to rather than an erosion or osmotic pumping‐controlled release there is a diffusion‐controlled release mechanism through the polymer matrix thanks to the structure of the PEG‐PST nanoparticle.

### 2.4 Cell Internalization Study

**Figure 4:**
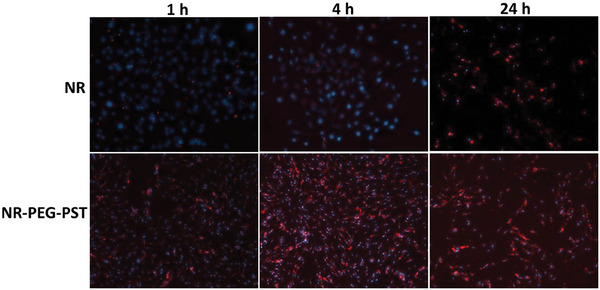
The intracellular uptake of free NR and NR‐PEG‐PST nanoparticles by A‐549 lung cancer cells after 1, 4, and 24 h treatment. Nuclei were stained with DAPI (blue). Nile Red exhibits red fluorescence.

The cellular uptake ability of prepared nanoparticles was determined using an A‐549 cell line after 1, 4, and 24 h. Nile Red (NR) a hydrophobic drug model was used by loading in PEG‐PST (NR‐PEG‐PST) nanoparticles or freely. Control group cells not treated with NR or NR‐NP were also prepared. The cellular uptake images for NR and NR‐PEG‐PST treated cells are presented in **Figure** **4**.

Fluorescence microscope images showed that higher fluorescence (red color) was observed for the NR‐PEG‐PST group compared to the NR group at the cytoplasmic level at all tested periods. In many cases, endocytosis and other cellular uptake mechanisms such as contact‐mediated cell internalization give nanostructures an advantage over small molecules.^[^ ^42^ ^]^ This is an important property of nanosized drug carriers as a versatile tool in drug delivery approaches. Similarly, NR‐PEG‐PST nanoparticles showed this effect which is proved by over‐increased red fluorescence in the NR‐PEG‐PST group in the cell internalization study (Figure 4).

**Figure 5:**
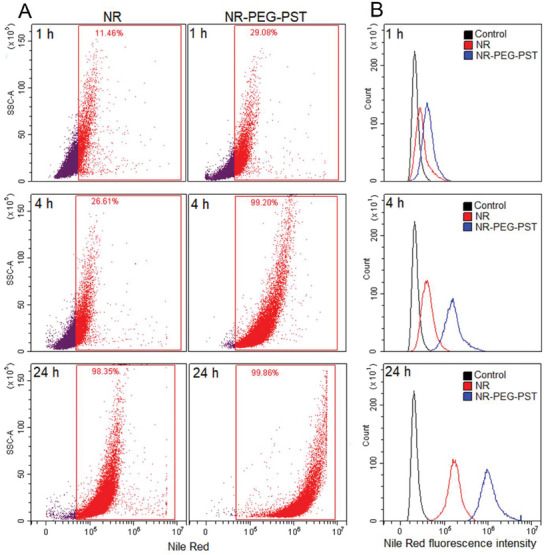
Flow cytometry analysis of NR and NR‐PEG‐PST internalization in A549 cells. A) Percentage ratio of the cells that internalized Nile Red. B) Mean fluorescence intensity of the cells in control, NR, and NR‐PEG‐PST groups after 1, 4, and 24 h treatment.

To find fount out quantitative results for the cell internalization study, the time‐dependent entry of Nile Red into the cells was investigated by flow cytometry (**Figure** **5**). In the study, the ratio of the A549 cells that internalized Nile Red (Figure 5A) and the mean fluorescence intensity values in the cells (Figure 5B) treated with NR and NR‐PEG‐PST were analyzed and compared with the control group after 1, 4, and 24 h of incubation periods.

At the 1st h of incubation, Nile Red internalization occurred in 11.46% of the cells in the NR group and 29.08% of the cells in the NP group. At the 4th h of incubation, Nile Red internalization occurred in 26.61% of the cells in the NR group and almost all of the cells (99.20%) internalized the fluorescent agent in the NR‐PEG‐PST group. At the 24th h of incubation, NR internalization occurred in 98.35% of the cells in the NR group and 99,86% of the cells in the NR‐PEG‐PST group (Figure 5A). Results showed that whereas almost all of the cells treated with NR‐PEG‐PST internalized the NR‐PEG‐PST at the 4th h, it was the 24th h in the case of the NR group. Besides these number‐based results, for a better understanding of the degree of internalization as a function of intensity, histogram values are shown for 1‐h, 4‐h, and 24‐h incubation periods (Figure 5B). According to the histogram values, NR‐PEG‐PST‐treated cells showed a rate of ten times higher fluorescent intensity (≈10^6^) than those treated with NR (≈10^5^). In summary, according to the number and intensity‐based flow cytometry results, NR‐PEG‐PST showed faster and higher cellular uptake than NR in A‐549 cells.

Amphiphilic nanoparticles are known to be internalized by cells via mainly three types of pathways depending on their size. These are clathrin‐mediated endocytosis for nanoparticles under 100 nm in diameter, caveolae‐mediated endocytosis for nanoparticles above 100 nm, and macropinocytosis for nanoparticles above 250 nm.^[^ ^43^ ^]^ Since PEG‐PST nanoparticles composed of amphiphilic PEGMEMA‐PST block copolymers ≈100 nm both clathrin and caveolae‐mediated endocytosis pathways are possible for cellular internalization. As a result of endocytosis, the release of loaded cargo accelerates due to the disassembly of amphiphilic block copolymer induced by lysosomal degradation.^[^ ^43^ ^]^ So, CBZ‐PEG‐PST nanoparticles are possible to disassemble and release CBZ faster in the cancer cells after lysosomal degradation causing higher cytotoxicity. The disassembled block copolymers can eventually be excreted from the body through the kidneys.^[^ ^44^ ^]^

### 2.5 Cytotoxicity of NPs

**Figure 6:**
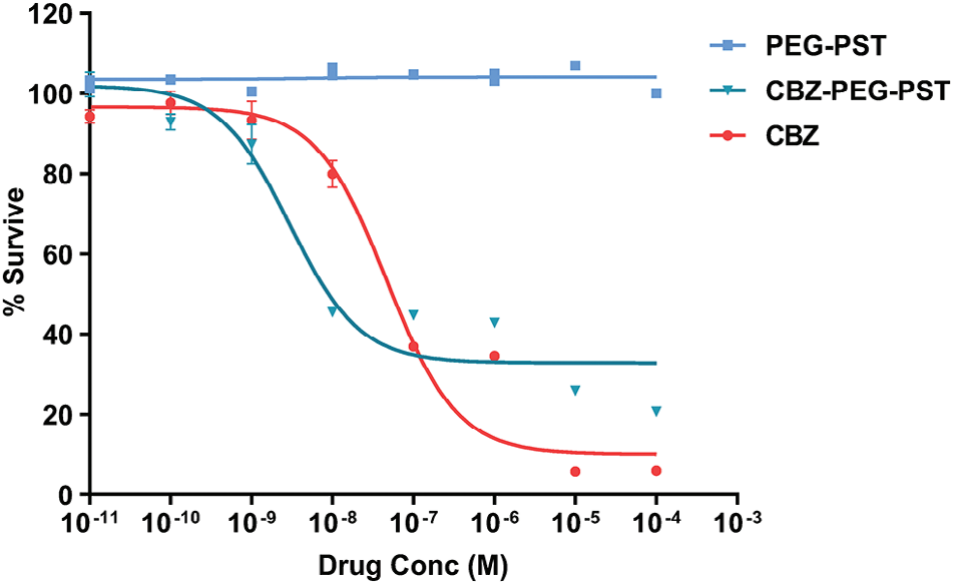
Cytotoxicity test of CBZ, CBZ‐PEG‐PST, and PEG‐PST in A549 cells using CCK‐8 assay. Data represent mean ± SD (*n* = 4).

PEG‐PST and CBZ‐PEG‐PST nanoparticles were evaluated regarding cytotoxicity compared to free CBZ in the A549 cell line. Since the CBZ‐PEG‐PST concentration samples were prepared as the CBZ equivalent of the free CBZ concentration samples, the drug concentration values in the graph show the CBZ concentration values of the CBZ and CBZ‐PEG‐PST group samples (**Figure** **6**).

On the other hand, since the PEG‐PST concentration samples were prepared to contain the same amount of PEG‐PST as the CBZ‐PEG‐PST group samples, the drug concentration values should be evaluated as the amount of PEG‐PST corresponding to CBZ in the CBZ‐PEG‐PST group. The CBZ‐PEG‐PST samples were prepared by serial dilution of PEGMEMA‐b‐PST nanoparticle solution containing 9.9% CBZ. So, it should be considered that the NP concentration values in both the CBZ‐PEG‐PST and PEG‐PST groups were ≈9 times the drug concentration indicated in the graph.

Drug delivery platforms are preferred to be designed with nontoxic vehicles.^[^ ^45^ ^]^ As a proof‐of‐concept model of this study, PEG‐PST nanoparticles prepared by PISA showed no toxicity at all tested concentrations. In addition to enabling the transport of hydrophobic drug molecules without the use of toxic solvents, nanocarriers have been widely used due to their superiority over free drugs in the uptake of drug molecules into cells by both endocytosis and contact‐ and receptor‐mediated intracellular delivery.^[^ ^42^ ^]^ This causes nanocarriers to be more cytotoxic to cancer cells than free drugs if they have ideal drug release profiles.^[^ ^46^, ^47^ ^]^ Similarly, in the study CBZ‐PEG‐PST group (EC_50_ = 4.05 × 10^−9^ m) showed higher cytotoxicity compared to the free CBZ group (EC_50_ = 4.67 × 10^−8^ m). When considering the cytotoxicity results together with the cell internalization study, higher cytotoxicity of CBZ‐PEG‐PST can be explained by superior cellular uptake over free CBZ. Moreover, the prolonged drug release profile of CBZ‐PEG‐PST must have been accelerated as a result of the unique lysosome environment (acidic pH, lysosomal enzyme activities (cathepsin B, sulfatase and b‐glucuronidase) in cancer cells,^[^ ^48^ ^]^ such that the CBZ‐PEG‐PST group was more effective than CBZ in cytotoxicity study with a period of 48‐h.

### 2.6 Lung Deposition Study (INH)

**Figure 7:**
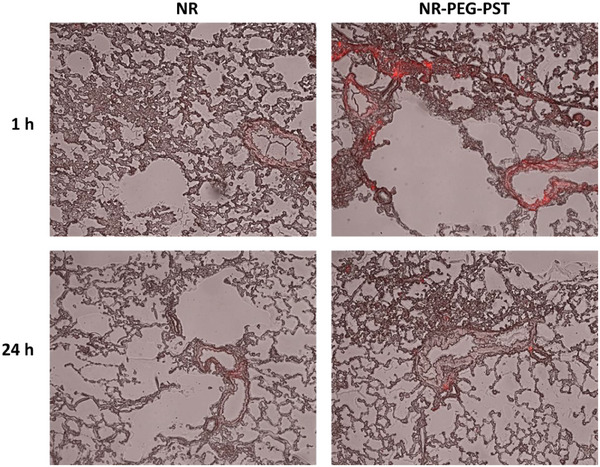
Lung deposition study of NR and NR‐PEG‐PST on rats. Merged bright field and red fluorescence images of frozen sections of rat lungs at 1 and 24 h after inhalation of NR and NR‐PEG‐PST. Nile Red exhibits red fluorescence.

The study investigated free Nile Red (NR) and NR‐PEG‐PST to understand their ability to accumulate in the lungs after nasal inhaler in rats. In inhalation, while the deposition of aerosols in the trachea and bronchi is explained by inertial impaction and gravitational force, respectively, the deposition of nanoparticles ≈100 nm in the deep lung region is reported to occur by diffusion as a result of Brownian motion.^[^ ^49^ ^]^ As a result of rapid systemic absorption,^[^ ^50^ ^]^ small free NR molecules were cleared very quickly compared to NR‐NP (≈100 nm) from deep lung region 1 h after inhalation in **Figure** **7**.

On the other hand, high red fluorescence was observed in the case of NR‐NP at the alveolar interstitium and vessel epithelium even 1 h after inhalation. 24 h after inhalation, while there was no sign of accumulation in the NR group, there was still red fluorescence in the alveolar interstitium in the NR‐PEG‐PST group. The decrease in red fluorescence in the NR‐PEG‐PST group after 24 h seems to have occurred due to alveolar macrophage clearance.^[^ ^50^ ^]^

The main reason why NR‐PEG‐PST accumulates for a longer time than NR is that the size of NR‐PEG‐PST is large enough to prevent diffusion through the capillary epithelium in the alveolar interstitium.

There are major differences between the absorption of nanosized particles and small molecules by diffusion and passing through the extracellular matrix barrier. In particular, the main parameter that controls the speed of passing through tissue‐specific barriers and capillary epithelial cells into the systemic circulation is size. In pulmonary delivery, small molecules are generally absorbed rapidly by diffusion and through active and passive transport through membranes after diffusing through the alveolar sacs, while nanoparticles remain longer due to their endocytosis pathways.^[^ ^51^ ^]^ Animal studies of inhaled nanoparticles revealed that only a small fraction of them applied translocate to the circulation and can reach other organs via the circulation.^[^ ^52^, ^53^, ^54^ ^]^ For this reason, various studies are being conducted on the use of various nanoformulations to provide higher lung bioavailability through inhalation for lung cancer.^[^ ^12^ ^]^ In a study, Hitzman and co‐workers formulated 5‐fluorouracil‐loaded lipid‐coated nanoparticles for the pulmonary route. Results showed that nanoparticles with lipid shells increased lung bioavailability, provided a sustained drug release in the lung, and reduced systemic toxicity.^[^ ^55^ ^]^ Kamel and co‐workers used lipid core‐protein shell nanoparticles for lung cancer dual therapy of Genistein and All‐Trans Retinoic Acid. The animal experiment showed that this inhalable nanoparticle formulation was superior to intravenously administered nanoparticle suspension against lung carcinoma. The formulation provided localized codelivery of Genistein and All‐Trans Retinoic Acid for lung cancer therapy.^[^ ^56^ ^]^ Raval and co‐workers formulated Silibinin‐loaded chitosan‐coated PLGA nanoparticles and showed that this nanoparticle strategy promoted to release of Silibinin in the lungs and increased vivo residence time in the lung.^[^ ^57^ ^]^

### 2.7 Histological Evaluation of NR and NR‐PEG‐PST Inhalation for Lung Tissue (INH)

**Figure 8:**
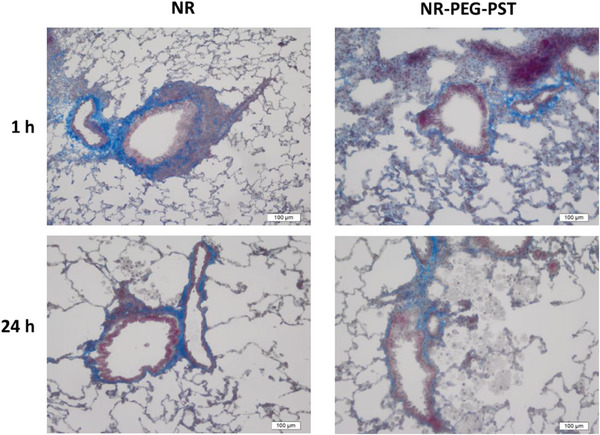
Crossmon's modified triple staining of paraffin sections of rat lungs at 1 and 24 h after inhalation of NR and NR‐PEG‐PST.

Histological analysis of rat lungs after NR and NR‐PEG‐PST inhalation at 1 and 24 h were evaluated on Crossmon's modified triple‐stained paraffin blocks of lung samples (**Figure** **8**).

In the histopathological evaluation of the lung tissues, it was clearly shown that alveolar macrophages highly increased in NR‐PEG‐PST inhaled rat's lungs at both 1 and 24 h after inhalation. On the other hand, there was a very low number of alveolar macrophages in the free NR group. When taken into account with the lung deposition study, it can be said that the alveolar macrophage response in the NR‐PEG‐PST group is due to the accumulation of nanoparticles in the deep lung region. Since alveolar macrophages are one of the major defense mechanisms of the immune system in the lungs against particles and microbes from the external environment,^[^ ^58^ ^]^ as a result of high nanoparticle accumulation in deep lung regions alveolar macrophage response was triggered. Although it is widely accepted that PEGylated nanoparticles are metabolized by alveolar macrophages after opsonization, Asoudeh et al.’s recent study showed that opsonization phagocytosis nexus is not the major factor driving PEGylated NP‐macrophage interactions. Instead, they suggested that PEG‐NP interactions with (apo)lipoprotein and scavenger receptors appear to be a major driving force for PEGylated NP binding, entry, and degradation in alveolar macrophages.^[^ ^59^ ^]^ Both hypotheses explain how NR‐PEG‐PST decreased after 24 h in the lung deposition study.

Asoudeh et al. also revealed that PEGylated nanoparticles or PEGylated liposomes did not increase or reduce the pro‐inflammatory cytokines and chemokines, unlike other opsonized pathogens.^[^ ^58^ ^]^ For this reason, it is expected that PEG‐PST nanoparticles with a PEGylated structure would not similarly cause inflammation.

The lung defense mechanism protects the lungs with methods such as enzymatic degradation and alveolar macrophage clearance after long‐term exposure to dust and other foreign substances.^[^ ^7^ ^]^ Therefore, it is thought that these proposed inhalable nanoformulations, which use biocompatible nanoparticles and can be applied with short‐term applications, will be well tolerated in the long term.

### 2.8 Biochemical Evaluation of Free CBZ and CBZ‐PEG‐PST (IV and INH)

Inhalation of drug‐loaded nanoparticles was evaluated in terms of biochemical and toxicological aspects in comparison with free drug inhalation and IV‐injected free and nano‐drug administrations. For this purpose, four groups were formed by administering both free Cabazitaxel (CBZ) and CBZ‐PEG‐PST via INH and IV routes. These are; CBZ (IV), CBZ‐PEG‐PST (IV), CBZ (INH) and CBZ‐PEG‐PST (INH).

**Figure 9:**
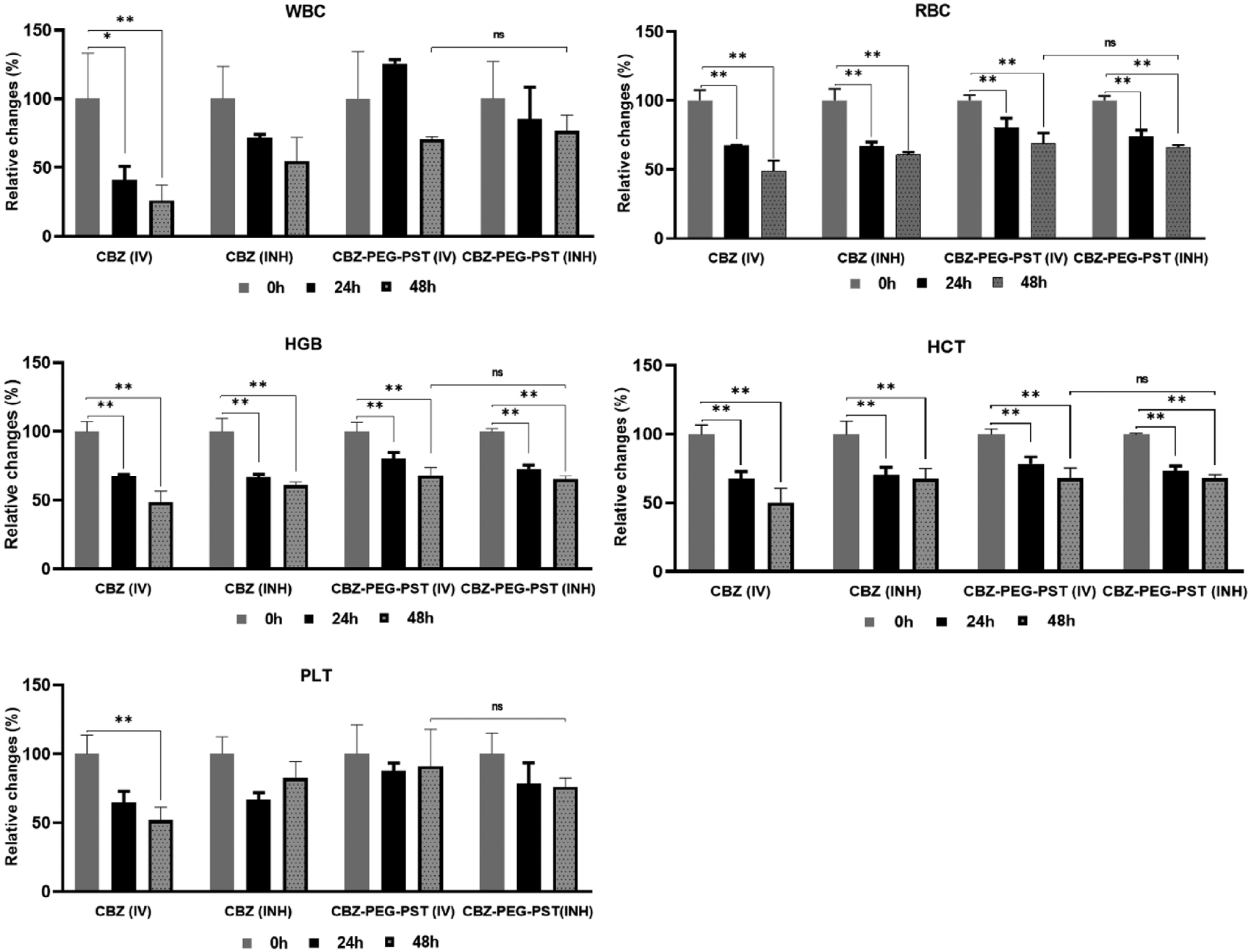
Hematological tests. Percentage changes in WBC (White Blood Cell), RBC (Red Blood Cell), PLT (Platelet), HGB (Hemoglobin), and HCT (Hematocrit) depending on time (0, 24, and 48 h). Data represent mean ± SD (*n* = 4). Differences between groups were considered significant at ^*^ *p* < 0.01 and ^**^ *p *< 0.001.

White blood cell (WBC), Red blood cell (RBC), Hemoglobin (HGB), Hematocrit (HCT), and Platelet (PLT) values ​​of the blood samples taken before dosing (blank), 24th and 48th h from the rats in these four groups are shown in **Figure** **9**.

As a result of traditional chemotherapy, hematological values such as WBC and RBC decrease significantly due to the toxicity of chemotherapy agents.^[^ ^60^ ^]^ This is the main reason why traditional chemotherapy fails in most cases. Although chemotherapy agents are successful in vitro, toxic effects prevent exceeding a certain dose in the clinic. As well as low treatment efficacy this situation causes the development of drug resistance.^[^ ^61^ ^]^

The nano drug formulations play a major role in decreasing systemic side effects by slowing down the release profile and making loaded drugs inactive in the systemic circulation.^[^ ^62^ ^]^ In an in vivo study, Yang and coworkers (2020) showed that CBZ‐loaded liposomes were significantly better tolerated (24‐fold of the tolerated dose) than the free CBZ formulation.^[^ ^63^ ^]^ Hematological test results in Figure 9, showed that the CBZ‐PEG‐PST group was better tolerated than the free CBZ group in both IV and INH applications according to WBC, RBC, and PLT results. In addition, the WBC value, which decreased by 75% in the free CBZ (IV) group at 48 h, decreased by 50% in the CBZ (INH) group during the same period. There was no significant difference between these two groups regarding RBC values. However, when the PLT values were compared, there was a 50% decrease in the CBZ (IV) group at 48 h, while this rate was 20% for the CBZ (INH) group. When these data in the IV and INH administration of the free CBZ are also considered, it is seen that the CBZ (IV) group is the least tolerated group among all groups even regarding the CBZ (INH) group. These data confirm information emphasizing that free IV drug administration is more toxic than local therapies and encapsulation strategies. In pulmonary drug applications, the amount of free drug passing into the systemic circulation is lower than in IV applications as a result of lung defense mechanisms. This situation was also clearly shown in the study by Borghardt and coworkers, where they examined olodaterol in IV and INH applications from a pharmacokinetic perspective.^[^ ^64^ ^]^ Similarly, in our study, this is the main reason why the free drug was tolerated the least after IV administration.

On the other hand, CBZ‐PEG‐PST was not toxic in the IV and INH‐applied groups and there was no significant difference between them. This shows that the effect of nano drug formulations in reducing systemic toxicity in IV applications is also valid in INH applications. To summarize, hematological results (Figure 9) show that the CBZ‐PEG‐PST formulation is better tolerated than free CBZ when administered both IV and INH, in terms of toxicity.

In our previous study, Kaga et al. investigated the biodistribution of radiolabeled PEG‐PST nanoparticles on tumor‐bearing nude mice.^[^ ^24^ ^]^ In the study, different‐sized spherical (diameter 21 and 33 nm), rodlike (diameter 37 nm, contour length 350–500), and worm‐like (diameter 45 nm, contour length 1–2 µm) nanoparticles were compared. As the nanoparticle size increased, the liver and spleen accumulation increased due to first‐pass elimination. Therefore, the best tumor accumulation was provided with the 21 nm nanoparticle. The results show that nanostructures above a certain size cannot be sufficiently accumulated in the tumor due to first‐pass elimination. It is also understood that the IV administered these nanoparticles did not show a specific distribution to the lungs. Similarly, many other studies have shown that the size effect is also valid for other nanoparticle systems in IV applications and the increase in size causes accumulation in organs such as the liver and spleen and thus reduces their blood half‐life and disables to reach the desired level of targeting in the tumor by showing a weak EPR (Enhanced Permeability and Retention) effect.^[^ ^65^, ^66^, ^67^ ^]^ Therefore, it is thought that this first‐pass elimination is avoided by administering PEG‐PST nanoparticles obtained with a size of ≈100 nm via the INH route in the current study.

**Figure 10:**
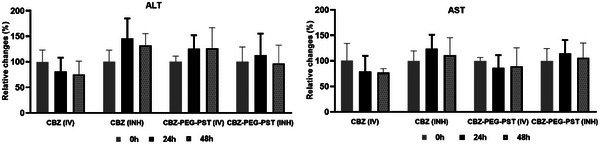
Serum ALT and AST values of rats treated with CBZ and CBZ‐PEG‐PST through IV or INH routes, depending on time (0, 24, and 48 h). Data represent mean ± SD (*n* = 4). Differences between groups were considered significant at ^*^ *p* < 0.01 and ^**^ *p *< 0.001.

Blood samples taken from CBZ (IV), CBZ‐PEG‐PST (IV), CBZ (INH), and CBZ‐PEG‐PST (INH) groups were also compared in terms of Alanine transaminase (ALT) and Aspartate transaminase (AST) values (**Figure** **10**). The 2 mg kg^−1^ free CBZ dose determined as the upper dose that can be reached in the inhalation formulation was kept constant in both the IV formulation and the CBZ‐PEG‐PST formulations to avoid variable parameters in the study. In other words, considering that a single dose of 2 mg kg^−1^ free CBZ or 2 mg kg^−1^ CBZ equivalent CBZ‐PEG‐PST was administered in both IV and INH applications, it was predicted that it would not cause significant toxicity in the liver and other organs.

As predicted, when blood samples taken from rats before dosing were compared with blood samples taken at 24 and 48 h, no statistically significant difference was obtained in ALT and AST values in all groups.

## 3 Conclusion

Chemotherapy drugs administered to the systemic circulation reach the target tissue in very small amounts after first‐pass elimination and due to the side effects, that occur as a result of direct administration to the circulation, cancer treatments are often not completely successful. However, INH is a second option in the treatment of lung cancer, apart from the IV route. Direct contact of the lung with the environment through the respiratory tract offers an alternative way for the treatment of lung cancer. The controlled drug release profiles of nano drug formulations and their accumulation properties in physiological barriers resulting from their sizes make them ideal drug carriers. In this context, we developed a PEG‐PST nano drug delivery system as an alternative to free drugs in our proof‐of‐concept design. These PEGylated nanoparticles were preferred due to their prolonged drug release profiles of up to 10–15 days determined by release studies and their nontoxic properties determined in in vitro tests. Our study with the loaded versions of Nile Red (NR‐PEG‐PST), a fluorescent agent, revealed that these PEGylated nanostructures provided higher NR accumulation in the lungs and longer retention up to 24 h compared to free NR. An in vivo study was also conducted to determine the differences in CBZ‐loaded versions (CBZ‐PEG‐PST) compared to free CBZ in IV and INH administrations. While the nano CBZ‐PEG‐PST formulations were found to be much better tolerated in both IV and INH administrations, WBC values were decreased by 75% in the CBZ(IV) group. Different parameters need to be tested in tumor‐bearing animals to prepare PEG‐PST nanoformulations with minimized toxic effects and optimized lung and tumor penetration. Due to the advantages provided by the lung defense mechanisms and the PEGylated nano drug delivery system, inhalable nano drug formulations were found to be more effective in targeting lung cancer and reducing the systemic side effects caused by chemotherapy drugs.

## 4 Experimental Section

### Materials

Oligo ethylene glycol methyl ether methacrylate (OEGMEMA) (M_n_: 300 g mol^−1^), chain transfer agent (CTA) 2‐Cyano‐2‐propyl benzodithioate, 2,2′‐Azobis(2‐methyl propionitrile) (AIBN), Styrene and Phosphate buffered saline (PBS) tablets were obtained from Sigma‐Aldrich. All solvents were HPLC grade, purchased from Merck, and used without purification. The human lung carcinoma (A549) cell line was obtained from the American Type Culture Collection (ATCC). Cell counting kit‐8 (CCK‐8), RPMI‐1640, and Fetal Bovine Serum (FBS) were obtained from Thermo Fisher Scientific.

### Synthesis of PEGMEMA

PEGMEMA polymer was synthesized using Reversible Addition‐Fragmentation chain Transfer (RAFT) polymerization.^[^ ^25^ ^]^ To a solution of OEGMEMA (1000 mg, 3.3 mmol) and 2‐Cyano‐2‐propyl benzodithioate (22.2 mg, 0.10 mmol) in methanol (2.5 mL) was added AIBN (1.6 mg, 0.01 mmol). The polymerization solution was purged with nitrogen to remove oxygen and the polymerization reaction was continued for 12 h at 70 °C under a nitrogen atmosphere in a sealed flask. The polymerization flask was cooled and unsealed to stop the reaction. Methanol was evaporated and crude was dissolved in 1 mL dichloromethane. Then the crude was precipitated in cold diethyl ether and waited for 6 h at −20 °C. Diethyl ether was discarded and crude was precipitated twice again after dissolving in 1 mL dichloromethane. After evaporation, the final precipitate waited 16 h in a high vacuum line to give PEGMEMA polymer. The molecular weight and PDI value of the PEGMEMA polymer were measured with Gel Permeation Chromatography (Shimadzu, THF‐based) using Polymethyl Methacrylate (PMMA) standards.

### Synthesis of PEG‐PST Nanoparticles by Polymerization Induced Self‐Assembly (PISA)

For the synthesis of the PEG‐PST nanoparticles literature protocol was applied by the PISA approach.^[^ ^24^ ^]^ To a solution of PEGMEMA as macro‐CTA (350 mg, 0.04 mmol) and styrene (ST) (20000 mg, 192 mmol) in methanol (28 mL), was added AIBN (1.23 mg, 0.0075 mmol). The reaction mixture was purged with nitrogen to remove oxygen. The reaction was started at 70 °C under a nitrogen atmosphere in a sealed flask to give Poly Styrene (PST) segment of PEGMEMA‐PST block copolymer. The newly formed PEGMEMA‐PST amphiphilic copolymer self‐assembled due to the insolubility of the PST segment in methanol, which was observed by the formation of turbidity. The reaction was stopped after 12 h by cooling and unsealing the flask. The formation of PEG‐PST nanoparticles was illustrated in Scheme 1. The milky white and turbid reaction solution was further dialyzed with cellulose dialysis tubing (Merck, 12.400 MWCO) against methanol to purify nanoparticles for 16 h (by refreshing the methanol every 4 h). The final concentration of the obtained nanoparticle solution was calculated by measuring the weight of the nanoparticle after evaporation and drying of 1 mL nanoparticle solution.

### Transmission Electron Microscope (TEM) Imaging and Dynamic Light Scattering (DLS) Analysis of Nanoparticles

Before TEM and DLS analysis methanol was removed from the nanoparticle solution. For this samples in methanol were dialyzed with cellulose dialysis tubing (Merck, MWCO: 12 400) respectively against methanol/water (8/2, v/v) for 2 h, methanol/water (6/4, v/v) for 2 h, methanol/water (4/6, v/v) for 2 h, methanol/water (2/8, v/v) for 2 h, and finally against water for 6 h (by refreshing the water every 3 h). Then nanoparticle solution samples in water (10 µL) were dropped on TEM grids and allowed to dry overnight at room temperature. Morphologies of the nanoparticles were analyzed using a JEOL JEM 1220 Transmission Electron Microscope with an acceleration voltage of 100 kV. Representative regions of nanoparticle samples were visualized at magnifications of 20 000. The hydrodynamic volume of nanoparticles was measured using a dynamic light scattering (DLS) instrument (Malvern Zetasizer Nano ZS).

### Nile Red (NR) and CBZ Loading of Nanoparticles

The post‐synthesis approach^[^ ^26^, ^27^ ^]^ was applied for loading the hydrophobic CBZ and NR into PEG‐PST nanoparticles. For CBZ loading, a solution of CBZ (18 mg/600 µL THF) was added to the nanoparticle solution (156 mg/6 mL) in methanol, and the flask was sealed. The flask was shaken in an orbital shaker for 2 h at room temperature. Then the mixture in the flask dialyzed with cellulose dialysis tubing (Merck, MWCO: 12 400) respectively against methanol/water (8/2, v/v) for 2 h, methanol/water (6/4, v/v) for 2 h, methanol/water (4/6, v/v) for 2 h, methanol/water (2/8, v/v) for 2 h, water for 3 h and finally against PBS buffer (pH: 7.4) for 3 h. For NR loading the solution of NR (3 mg/600 µL THF) was added to the nanoparticle solution (156 mg/6 mL) in methanol, and the flask was sealed. The flask was shaken in an orbital shaker for 2 h at room temperature. NR loading was followed similarly to the CBZ loading procedure. The amount of loaded CBZ and NR in nanoparticles was determined by UV–vis Spectrophotometer (Shimadzu UV‐1280) via CBZ and NR calibration curves to calculate loading efficiency. The calibration curves were obtained by plotting absorbance values as a function of the concentration of standard solutions of CBZ and NR at 277 and 532 nm, respectively. Percent Loading Content (LE) and Loading Efficiency (LE) values were calculated using the equations below.

(1) $$\begin{array}{cc} & \mathrm{Loading}\;\mathrm{Concent}\;(\%)=100\\ & \;\times \frac{\mathrm{Weight}\;\mathrm{of}\;\mathrm{loaded}\;\mathrm{material}\;\mathrm{in}\;\mathrm{NP}}{\mathrm{Weight}\;\mathrm{of}\;\mathrm{loaded}\;\mathrm{material}\;\mathrm{in}\;\mathrm{NP}+\mathrm{Weight}\;\mathrm{of}\;\mathrm{NP}} \end{array}$$

(2) $$\begin{array}{cc} \mathrm{Loading}\;\mathrm{Efficiency}\;\left(\%\right) & =100\\ & \;\;\times \frac{\mathrm{Weight}\;\mathrm{of}\;\mathrm{loaded}\;\mathrm{material}\;\mathrm{in}\;\mathrm{NP}}{\mathrm{Weight}\;\mathrm{of}\;\mathrm{material}\;\mathrm{added}} \end{array}$$

### CBZ and NR Release

Release studies were immediately performed after the CBZ and NR loading stages according to literature procedures.^[^ ^28^, ^29^, ^30^ ^]^ CBZ and NR‐loaded PEG‐PST nanoparticle solutions (3 replicates, 2 mL each) were placed in Pur‐A‐Lyzer Maxi Dialysis Kit (Merck, MWCO: 12 000). Then the kits were immersed in 20 mL release medium (PBS buffer (pH: 7.4) including 10% ethanol, 0.1% Tween 80) at 37 °C on an orbital shaker with 100 RPM. 2 mL release samples were taken from the release mediums and the amount of released CBZ and NR were measured respectively at 277 and 532 nm at certain time points using UV–vis Spectrophotometer (Shimadzu UV‐1280) via CBZ and NR calibration curves. The release samples were transferred to the release mediums immediately after the measurements.

### Cell Culture

The human lung cancer cell line (A549) was used for in vitro studies. The cell line was cultured in 10% FBS, 100 U mL^−1^ penicillin‐streptomycin supplemented Roswell Park Memorial Institute 1640 (RPMI‐1640). Cells were incubated at 37 °C, 5% CO_2_.

### Cell Internalization of NPs

Cell internalization analyses of nanoparticles were evaluated using fluorescence microscopes. Cancer cells were seeded with 5 × 10^4^ cells per well. The cells were incubated for 24 h for adhesion. After the adhesion of the cells, the prepared free NR and NR‐loaded nanoparticle solutions (10 µm, 1 mL of medium) were applied to the cells for 1, 4, and 24 h of incubation. Cells were visualized using an inverted fluorescence microscope (Zeiss Axio Observer Z1) and Zen Blue Edition software. NR and DAPI were excited using 560 and 365 nm lasers, respectively.

Time‐dependent cell internalization profiles of free NR and NR‐loaded PEG‐PST nanoparticles were analyzed using a flow cytometer. Cancer cells were seeded with 5 × 104 cells per well. Prepared free NR and NR‐NP solutions (10 µm,1 mL medium) were applied to the cells and the samples were incubated for 1, 4, and 24 h. After incubation, the cells were washed twice with 1x PBS. Following this procedure, fixation was performed with 4% PFA, and the permeabilization was performed with 0.25% Triton‐X100 for 20 min. Analysis was done in the FL‐2 channel (Beckman, DxFLEX). Image analyses were performed using CytExpert software.

### Cytotoxicity Test

In vitro, cell cytotoxicity tests were performed in the A549 cell line using a CCK‐8 test kit. Three groups; CBZ, CBZ‐PEG‐PST, and PEG‐PST were tested. CBZ samples were prepared at different CBZ concentration values between 10^−11^ and 10^−4^ M. CBZ‐PEG‐PST samples with the same CBZ quantities (CBZ equivalent) were also prepared. PEG‐PST nanoparticle group samples containing the same PEG‐PST nanoparticle amount as the CBZ‐PEG‐PST group samples were also prepared. Cells (3 × 10^3^) were seeded in 96‐well plates with three replicates for each sample. After overnight incubation, the medium of the cells was replaced with a medium containing increasing concentrations of free drug and drug‐loaded nanoparticles. After 48 h of incubation, the medium was removed and the wells were washed with 1xPBS. To determine cell viability, 100 µL of medium containing 10% CCK‐8 solution was added to each well, and absorbance values were determined at 450 nm using a plate reader at the end of 4 h of incubation. The viability of drug‐applied cells was expressed as a percentage of control cells using GraphPad Prism 8.0 software (La Jolla, CA, USA).

### Animals

*Male* Wistar‐Albino rats (270–320 g) were obtained from Afyon Kocatepe University Experimental Animals Application and Research Center and maintained in a controlled environment with a 12 h light/dark cycle. Water was provided ad libitum. After internal jugular vein and carotid artery catheterization rats were allowed to recover in metabolism cages for 24 h with harness jackets. Food was withheld for 8 h for pre and postdosing but was provided ad libitum at all other times. Animal experiments were approved by the Afyon Kocatepe University Animal Experiments Local Ethics Committee (Approval number: 49533702/286).

### Internal Carotid Artery and Jugular Vein Catheterization

The male Wistar Albino rats used for IV administration were catheterized via the internal carotid artery and jugular vein according to the literature protocol.^[^ ^31^ ^]^ The animals were given ad libitum water for 8 h the night before this procedure was performed and the rats were left fasting. The rats were deeply anesthetized with isoflurane before the surgery. Local anesthesia was also applied to the surgical areas with prilocaine HCl. After the internal carotid artery and jugular vein catheterization, the catheters were passed subcutaneously and removed from the nape of the neck. The surgical areas were sutured closed. The rats were dressed in specially prepared jackets and the catheters were passed through the steel spiral channel fixed to these jackets. After the surgery, the rats were taken to their metabolic cages. The steel spiral channel was fixed on the cage. No anesthesia was applied to the rats during dosing and blood sampling.

### Lung Penetration Study (INH Administration)

NR was dissolved with 13% v/v solution of ethanol in saline (1 mL) to give free NR solution (0.4 mg kg^−1^). Freshly prepared NR‐PEG‐PST solutions (0.4 mg kg^−1^ NR equivalent 2% NR loaded NR‐PEG‐PST, 1 mL) were used for dosing. Four groups (*n* = 3 for each group) were created for free NR and NR‐PEG‐PST dosing, NR (INH)‐1 h, NR‐PEG‐PST (INH)‐1 h, NR (INH)‐24 h, and NR‐PEG‐PST (INH)‐24 h. For INH administration, rats were restricted in a nose head‐only device, then NR (0.4 mg kg^−1^, 1 mL) and NR‐PEG‐PST (0.4 mg kg^−1^ NR equivalent 2% NR loaded NR‐PEG‐PST, 1 mL) solutions were dosed using a nebulizer in 5 min. Rats in NR (INH)‐1 h, NR‐PEG‐PST (INH)‐1 h groups were sacrificed at 1 h and rats in NR (INH)‐24 h and NR‐PEG‐PST (INH)‐24 h groups were sacrificed at 24 h after dosing by exsanguination under anesthesia. Collected lung samples of the rats were embedded in a tissue embedding matrix (OCT) then waited in a −20 °C freezer for 3 h and transferred to a −80 °C freezer till fluorescence microscope imaging and histological analysis.

### Fluoresce Microscope Imaging

Lungs were removed from the rats and after inflating the lungs with optimum cutting temperature (OCT) compound whole lungs were immersed in a mold containing OCT compound. Then, lung samples were quickly transferred to ice and kept at −80 °C until sectioning. Sections were taken from the prepared lung tissues with a cryostat microtome device (Leica CM1860, United States) and placed on positively charged slides. The borders around the tissue sections were drawn with an ImmunoEdge Pen and waited to dry. Then, a washing process was carried out with 1XPBS to cover the section surfaces. After washing, section surfaces were incubated with DAPI (1:1000 in 1XPBS) for 5 min. Then, the tissue sections were washed three times with 1X PBS for 5–10 min and visualized under a fluorescent microscope (Zeiss Axio Observer Z1) and Zen Blue Edition software. NR and DAPI were excited using 560 and 365 nm lasers, respectively.

### Method for Paraffin Blocks

The lungs were removed and fixed in 10% neutral buffered formalin for 2 days and the tissues were washed with tap water, dehydrated through 70, 80, 95, and 100% alcohol, cleared in two baths of xylene, and embedded in paraffin. The sections (5 µm thickness) were cut from the paraffin blocks and placed on the slides. The sections were stained with triple stain according to the literature.^[^ ^32^ ^]^ The slides were examined under an Olympus BX50 microscope.

### Biochemical Evaluation of Free CBZ and CBZ loaded NPs (IV and INH Administration)

CBZ in 125 µL Tween 80 was dissolved with 875 µL 13% v/v solution of ethanol in saline to give 1 mL dosing solution (2 mg kg^−1^) for Free CBZ.^[^ ^33^ ^]^ Freshly prepared CBZ‐PEG‐PST nanoparticle solutions (2 mg kg^−1^ CBZ equivalent, 10% CBZ loaded CBZ‐PEG‐PST /1 mL) were used for dosing. These two formulations were used for both IV and INH routes. Four groups (*n* = 4 for each group) were created for free CBZ and CBZ‐PEG‐PST dosing, CBZ (IV), CBZ‐PEG‐PST (IV), CBZ (INH), and CBZ‐PEG‐PST (INH). In the case of the IV administration route, CBZ (2 mg kg^−1^, 1 mL) and CBZ‐PEG‐PST (2 mg kg^−1^ CBZ equivalent, 10% CBZ loaded CBZ‐PEG‐PST /1 mL) solutions were dosed via jugular vein cannulas in 5 min in metabolism cages. In the case of the INH administration route, rats were restricted to nose head‐only device, then CBZ (2 mg kg^−1^, 1 mL) and CBZ‐PEG‐PST (2 mg kg^−1^ CBZ equivalent, 10% CBZ loaded CBZ‐PEG‐PST /1 mL) solutions were dosed using nebulizer in 5 min. Blood samples were collected just before dosing (blank), 24 and 48 h. At the end of the 48th h, rats were sacrificed by exsanguination under anesthesia, and then lungs, liver, heart, spleen, and kidneys were collected.

### Analysis of Blood Samples

Blood samples (200 µL) for Complete Blood Count (CBC) were immediately analyzed, whereas blood samples (200 µL) for biochemical analysis were centrifuged for 3 min at 6000 RPM, and obtained plasma samples were kept at −20 °C till analysis. CBC analysis was performed using a hematology analyzer (Sysmex XN‐1000, Japan), and hemogram values of rats for blank, 24, and 48 h blood samples were measured. Biochemical analysis was performed using a biochemistry autoanalyzer (Roche Cobas 8000 ISE Module Analyzer, Germany), and ALT and AST values of rats for blank, 24 and 48 h blood samples were measured.

### Statistical Analysis

Results of measurements and experiments were recorded as means ± SD. The statistical analyses were performed by the GraphPad Prism 8.0 software using two‐way ANOVA followed by multiple comparisons by post hoc Tukey's test (alpha = 0.05). The differences between groups were considered significant at ^*^ *p* < 0.01 and ^**^ *p *< 0.001. All in vitro and in vivo experiments were studied at least in replicates.

## Conflict of Interest

The authors declare no conflict of interest.

## Data Availability

The data that support the findings of this study are available from the corresponding author upon reasonable request.
